# Effects of Low-Frequency Electromagnetic Field on the Physicochemical Properties of Freeze–Thawed Mongolian Cheese

**DOI:** 10.3390/foods12081567

**Published:** 2023-04-07

**Authors:** Xueyan Yun, Yawen Deng, Yangyang Wang, Yueyuan Lu, Tungalag Dong

**Affiliations:** College of Food Science and Engineering, Inner Mongolia Agricultural University, Hohhot 010018, China

**Keywords:** low-frequency electromagnetic field, freeze–thawed Mongolian cheese, texture quality, microstructure, rheological properties

## Abstract

To verify whether a low-frequency electromagnetic field (LFE field) can help reduce structural damage during the freeze–thaw process and maintain shelf life, Mongolian cheese was frozen at −10, −20, and −30 °C, then thawed at microwave or room temperature. Results showed that LFE field-assisted frozen treatment could reduce ice crystal size and protect the protein matrix structure of cheese. Frozen–thawed cheese retained 96.5% of its hardness and showed no significant difference from the fresh one in elasticity, cohesion, and chewiness. Frozen cheese showed similar but slower ripening behavior during storage, suggesting a potential application of the LFE field in the frozen storage of high-protein foods.

## 1. Introduction

Mongolian cheese is a kind of traditional dairy food in Inner Mongolian, which is nutritious but difficult to preserve [1]. The making procedure of this cheese starts with naturally fermented sour milk, which is degreased and then heated to separate the whey. During this process, it is continuously stirred until the viscosity of the milk protein increases and wiredrawing begins, and then loaded into a mold for molding. Similar to common cheese ingredients, it is rich in protein [2]. At present, freezing is the most used preservation method in extending the shelf life of cheese, but it causes deterioration of water retention and hardness. It has been reported that frozen Spanish goat cheese has a stronger graininess, and a sour taste accompanied by an oxidized taste after three months of storage [3]. Both macroscopic and microscopic changes occur, which is also reflected in rheological properties. The elasticity of frozen cheese will decrease with the extension of storage time [4]. The decline in quality is mainly because ice crystals will expand the gaps in the structure. Some needle-shaped ice crystals will even pierce the connection inside of the matrix network [5]. Some strategies have been carried out to improve the quality of frozen cheese. The shelf life of frozen mozzarella cheese with a protective solution is extended to more than 60 days [6]. Higher salt concentration can reduce the damage caused by freeze–thaw cycles of cheese, but increase the hardness. Blast freezing technology has also been applied to reduce the size of ice crystals and improve cheese storage quality [7].

A low-frequency electromagnetic field (LFE field) is an energy field with a frequency below 300 Hz, which can increase the supercooling temperature of water and induce ice crystal nucleation [8]. It has been reported that the LFE field-assisted freezing would cause water molecules to oscillate and inhibit the growth and even change the shape of ice crystals [9]. Recently, electric and magnetic fields have been used in the pre-treatment of freeze-drying to give the product a finer taste [10]. The results showed that the electric field could increase the rate of nucleation, but had little damage to the meat fibers [11]. As a result of controlled nucleation, the juice loss of meat after thawing was significantly reduced. In addition, the electric field could maintain water-holding capacity and elasticity of the product [12]. Based on those studies, the electric field plays a positive role in the preservation of frozen food. However, few reports have focused on the legacy effect of its application in the preservation of frozen cheese.

At present, frozen–thawed cheese would have a slightly sour taste, and always lose elasticity and chewiness. The structure of cheese becomes less compact because of the structural collapse and freeze-concentration effects induced by the freeze–thaw process. In this study, the LFE field was used to minimize the size of ice crystals during freezing, aiming at maintaining the structure and the quality of frozen–thawed Mongolian cheese. The texture characteristics, rheological properties, microstructure, and sensory quality were recorded to evaluate the effect of the LFE field on the physicochemical properties of cheese.

## 2. Materials and Methods

### 2.1. Materials

Mongolian cheese was purchased from herders (Xilin Gol City, Inner Mongolia, China) and transported to the laboratory within 2 h in insulated bags. 

### 2.2. Methods

#### 2.2.1. Frozen Sample Preparation

The tops and bottoms of cheese cubes were carefully removed by a sharp knife and cut into small cubes of about 60 × 60 × 60 mm^3^ on a sterile operating table. Those samples were sealed separately in polyethylene bags and stored at −10, −20, and −30 °C for 24 h, with and without an electric field. Subsequently, one group of the frozen samples was removed from the freezer and thawed at room temperature (about 25 °C) for 4 h, with or without the LFE field. The other group was thawed with a microwave oven (EG720FA4-NR, Midea, Foshan, China, 2.45 GHz) and heated for 120 s with 100 W, which was set up as a control group (CK). All samples were coded as frozen/temperature-frozen/treatment-thawed treatment. The experimental design and name code explanations are shown in Table 1 and Table 2. Based on the optimum frozen–thawed method, a group of samples was frozen at −30 °C and stored for 14 days to observe the effect of the LFE field on amino acids and fatty acids during storage. In addition, the fresh cheese without any treatment was also refrigerated at 4 °C as the CK group. The sample names were coded with frozen/treatment-thawing/treatment-day of storage. For example, E-M-7 represents the sample that was frozen at −30 °C with an LFE field and then thawed by microwave after 7 days of storage. Usually, each test has three parallel samples, with each test repeated twice.

#### 2.2.2. Sensory Analysis

The sensory quality of the cheese was evaluated by 10 trained panelists. Descriptors were evaluated regarding the appearance (milky color, even surface, shiny), odor (milk flavor, butter, and unpleasant smell), taste (cheese flavor, acid, and bitter taste), and structure (hardness, fineness, elasticity), 20, 20, 25, and 35 points, respectively. Please refer to Table S1 for specific evaluation details.

#### 2.2.3. Microstructure Analysis

After being dehydrated in a graded acetone series (50, 75, 95, 100% (*v*/*v*)), the natural broken surfaces of the samples were examined by TM 4000 plus scanning electron microscope (SEM) at 3 kV with a working distance of 15 mm and a BSE signal mode.

#### 2.2.4. Dynamic Rheology

Three parallel samples were mixed and used for rheological measurements. Each test was repeated twice at 25 °C using a controlled stress rheometer (RS-6000, Haake Technik, Vreden, Germany). A stress sweep determination was performed on a different aliquot to ascertain linearity. Measurements were performed at a constant strain of 0.5% within the linear viscoelastic region, and all frequency sweeps were conducted with oscillation frequencies ranging from 1 to 10 Hz. 

#### 2.2.5. Textural Properties

Texture profile analysis was carried out by compressing the samples at a strain rate of 100 mm/min with six repetitions. The T-head was moved slowly until 50% deformation of thickness of the sample was reached. Texture parameters of the samples, including resilience, hardness, gumminess, and chewiness, were measured and calculated. 

#### 2.2.6. Amino Acid Analysis

Amino acid contents were determined by mixing six parallel samples in an amino acid analyzer (L-8900, Hitachi, Japan). A total of 1.0 g of crushed homogeneous samples was weighed and put into a hydrolytic tube. Then, 10 mL 6 mol/L hydrochloric acid and three drops of phenol were added. The tube was semi-frozen, vacuumed, and sealed with nitrogen. After being maintained at 110 °C for 22 h in a constant-temperature heating box, the hydrolysa was rinsed with deionized water several times and the volume was set to a 50 mL volumetric bottle. A total of 1 mL of liquid was distilled and dried at 40 °C under pressure, then 2 mL of water was added to dry again. The dry substance was dissolved in 2 mL 0.02 mol/L hydrochloric acid, and then injected into a sample bottle. After being treated with a 0.22 μm filter membrane, the sample was tested by amino acid analyzers. The chromatographic conditions were as follows: the packing of the separation column was 4.6 mm ID 60 mm l sulfonic acid cation exchange resin; the flow rate of pump 1 was 0.40 mL/min; the flow rate of pump 2 was 0.35 mL/min. The sample injection volume was 20 μL, and the analysis time for each cycle was 53 min with sodium citrate as a buffer, and each test was repeated twice.

#### 2.2.7. Fatty Acid Analysis

Fatty acid contents are tested by mixing six parallel samples with two receptions on a fatty acid detector (GCMS-QP2010UITRA, Shimadzu, Kyoto, Japan). The chromatographic conditions were as follows: the split ratio was 20:1, the initial temperature was 60 °C, and hold for 1 min, then heated to 140 °C at 40 °C/min, hold for 10 min, and then rise to 240 °C at 4 °C/min, hold for 15 min. Mass spectrometry conditions: ion source temperature was 200 °C, interface temperature was 240 °C, solvent delay time was 9.7 min, and scanning range was 50~500 *m*/*z*. The external standard method was used for the qualitative and quantitative analysis of fatty acids. The temperature of the injection port was 220 °C, and the injection volume was 1 μL. The carrier gas was helium, and the column flow was 1 mL/min.

#### 2.2.8. Methodology

Single-factor ANOVA using Minitab, α = 0.05. Both polynomial fitting curves and PCA analysis used Origin 2019b, with a second-order polynomial and a confidence level of 95%. The principal component extraction number was 2, and the analysis mode was the correlation matrix.

## 3. Results

### 3.1. Sensory Characteristics

**Figure 1:**
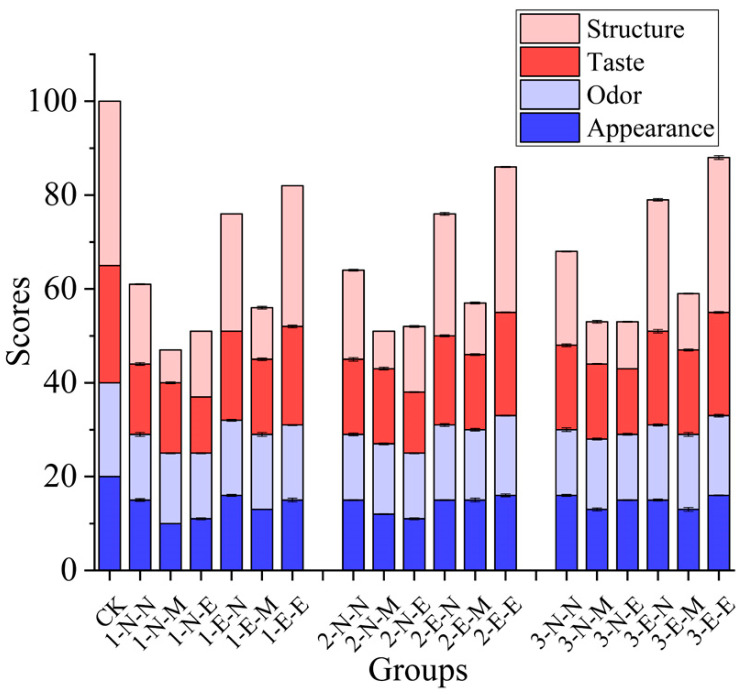
Sensory scores of freeze–thawed Mongolian cheeses.

Figure 1 shows the sensory scores of frozen–thawed cheeses. As shown in Figure 1, the unfrozen cheese has the highest sensory score due to its undamaged texture. After being frozen at −10 °C without any treatments, the frozen–thawed 1-N-N cheese showed a much lower sensory score of about 60 points. In the case of microwave-assisted thawed samples, the 1-N-M achieved the lowest sensory score of about 47 points in all groups. For the 1-N-E group, the cheese only achieved a slightly higher sensory score of about 50 points with LFE field-assisted thawed treatment, suggesting less effect of the LFE field during the thawing process. Compared with the 1-N-E group, the cheese of the 1-E-N group showed a much higher score of about 78 points due to the LFE field-assisted frozen treatment. For the 1-E-E group, cheese has a sensory score near 85 points since it was frozen and thawed with the LFE field. After being frozen at −20 and −30 °C, the sensory scores of cheeses showed a similar trend to that at −10 °C. It should be noted that the scores of the cheeses frozen and thawed with the LFE field (1,2,3-E-E) were all higher than 80 points, indicating better sensory quality. The cheese frozen without an LFE field assistant showed lower taste scores. The scores on the structural dimension of the samples thawed by microwave and LFE field are also lower than those frozen in the LFE field. The scores of other dimensions are also relatively lower but show little difference, which may indicate that the slow freezing speed and microwaves mainly cause the deterioration of the structure of the cheese. It is generally believed that although microwaves can improve the thawing rate, the damage caused by local overheating cannot be ignored. This uneven thawing phenomenon would harm the thermal stability and structure of proteins.

**Figure 2:**
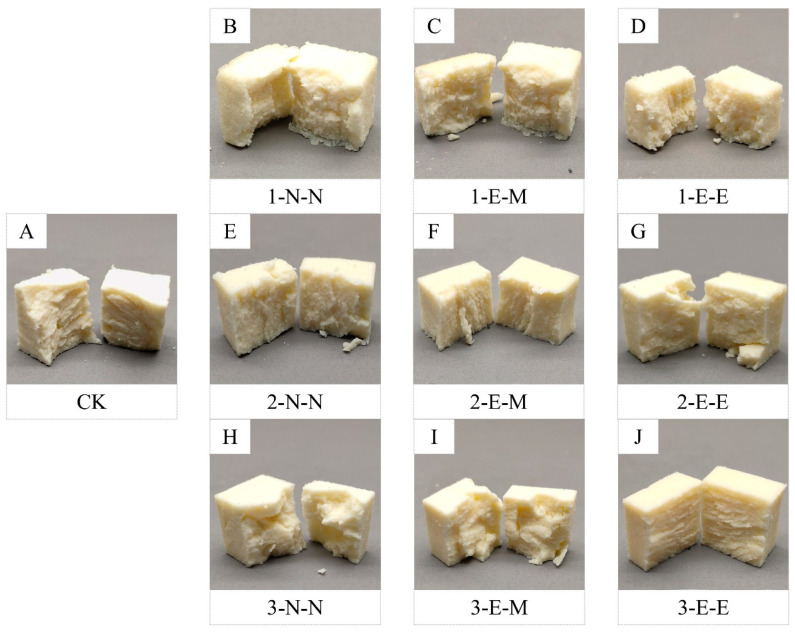
Photos of the surface and cross-section of freeze–thawed Mongolian cheeses.

Figure 2 shows the photos of the surface and cross-section of freeze–thawed cheese under different conditions. As shown in Figure 2A, the fresh cheese presents a neat section and shows no obvious crumbs under the action of external force. After being frozen at −10, −20, and −30 °C, the section of cheese directly thawed at room temperature (Figure 2B,E,H) presented different irregular sections with crumbs, suggesting a more fragile structure. It also can be seen that the lower freezing temperature brings a tighter structure to frozen–thawed cheese. With LFE field-assisted frozen treatment, the microwave-assisted thawed cheeses showed slightly neater and tighter sections with fewer crumbs (Figure 2C,F,I), which was very similar to the thawed cheese in Figure 2B,E,H. This may be due to the local overheating problem during the microwave thawing process [13]. When the core temperature is enough to melt the cheese, some parts have not been completely thawed and this uneven texture may make the cheese structure more unstable. As can be seen from Figure 2D,G,J, cheese kept a relatively neater and denser structure with the participation of the LFE field during the freeze–thawing process, suggesting that the LFE field plays a positive role in decreasing the undesirable impact caused by freezing storage. In addition, the cheese frozen at −30 °C in 3-E-E showed almost the same selection as fresh cheese, indicating that lower temperature also was favorable to the freezing preservation of cheese. The photo accorded well with the result of the sensory evaluation.

### 3.2. Microstructure Analysis

**Figure 3:**
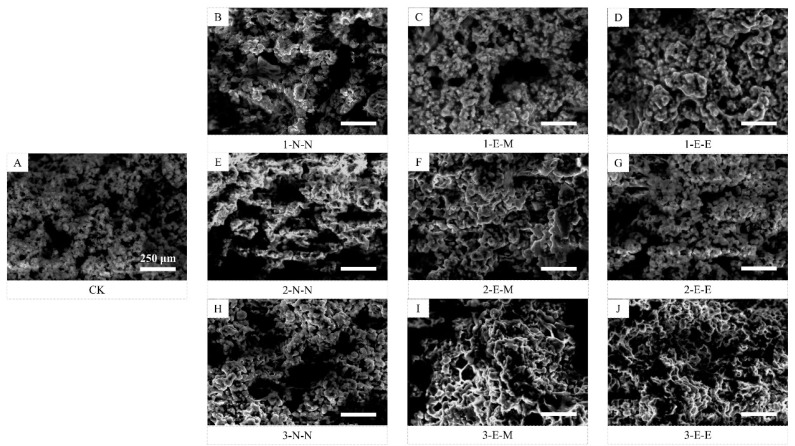
SEM images of freeze–thawed Mongolian cheese.

Figure 3 shows the SEM micrographs of cheeses. As shown in Figure 3A, fresh cheese presents a relatively uniform microstructure with small holes. Those holes may be due to the incomplete discharge of small bubbles after stirring and the absence of pressing in the manufacturing process. The amorphous protein matrix of cheese consists of easily recognizable structural units, including subcellular casein and micelles, fat globules, and very small starter bacteria. Subcellular casein has a diameter of 15–20 nm, a micelle of 50–300 nm, and milk fat globules have diameters between 0.1 and 10 mm [14]. It can be observed from Figure 3B,E,H that a certain agglomeration phenomenon and numerous big and deep void spaces appeared in the protein matrix of thawed cheese after being frozen at −10, −20, and −30 °C. It may be concluded that ice crystals were initially present in those spaces and removed during the freezing process. Damaged granules of micelles with a rough surface can be found in Figure 3B,E. In comparison to Figure 3B,E,H, the protein matrix of the cheese treated by LFE field-assisted freezing plus microwave-assisted thawing in Figure 3C,F,I had a much smoother surface with smaller and shallower void spaces, suggesting that smaller ice crystals had already existed before ice sublimation in 1-E-M, 2-E-M, and 3-E-M. In the case of LFE field-assisted freeze–thawed cheeses, the 1-E-E, 2-E-E, and 3-E-E not only had a uniform and much smoother protein matrix, but also had visible discernible and fibrous protein strands. In addition, several fat globules and round particles in a row between strands of protein fibers in the frozen samples were very distinguishable in contrast to cheese in the 1-E-M and 2-E-M, indicating more perfect protein networks and less damage of smaller ice crystals. There is a complex interaction between the microstructure, texture, and flavor of cheese [15]. The holes supported by ice crystals may provide more enzymatic reaction sites. With the decrease in thawing rate, the extension of time, or the thermal effect brought on by microwaves, the decomposition of the protein and other reactions in cheese were accelerated [16]. The existence of an LFE field promotes smaller crystals and indirectly accelerates the thawing rate, leading to more uniform and complete protein structures.

### 3.3. Dynamic Viscoelastic Behavior Analysis

**Figure 4:**
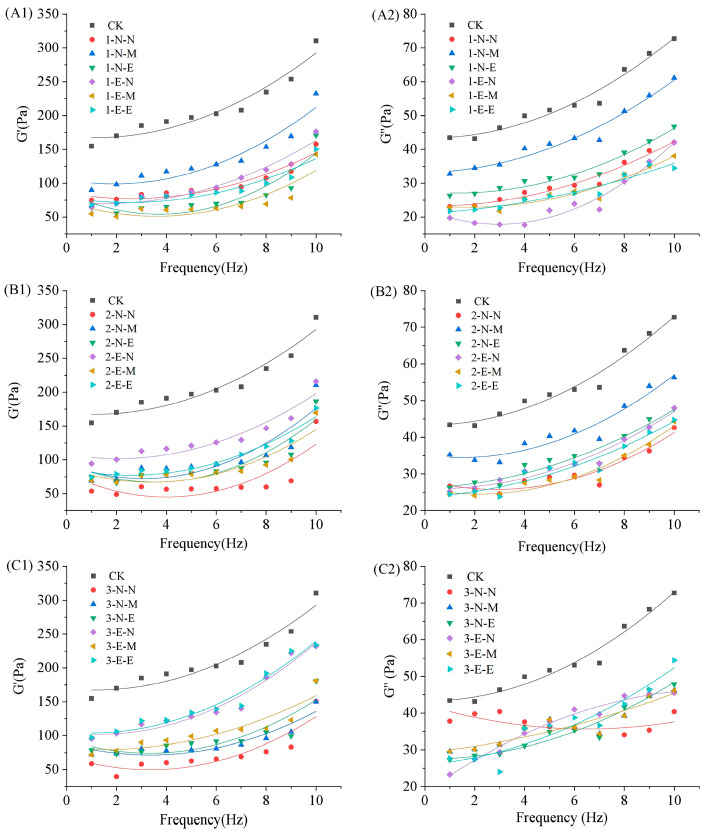
Rheological properties of freeze–thawed Mongolian cheese. (**A1**,**B1**,**C1**) are G’ plots of frozen cheese at different temperatures (−10 °C, −20 °C, −30 °C), respectively. Correspondingly, (**A2**,**B2**,**C2**) are G’ plots.

The result of viscosity measurements was expressed in terms of the elastic or storage modulus (G′) and viscous modulus (G″), which reflect the elasticity and viscosity properties of cheese. As shown in Figure 4, the G′ and G″ of all samples showed an increased tendency with increasing test frequency, indicating higher viscoelasticity at a higher frequency. This suggests that elastic behavior is more dominant than stickiness in both fresh and freeze–thawed cheeses. In addition, according to the power-layer model, elasticity is more susceptible to frequency changes [17]. It can be seen from Figure 4 that G′ and G″ of all freeze–thawed cheese were lower than the fresh cheese (CK group), suggesting a decreased viscoelastic of cheese. As shown in Figure 4(A1),(A2), the cheeses of 1-N-M have higher G′ and G″ values than all the other groups. This may be because larger crystals are easy to form during the slow natural freezing process. It takes a long time to microwave thaw, leading to a great loss of water in cheese. However, the effect of the LFE field on the other groups was not significant. With the freezing temperature decreased to −20 °C, the 2-E-N, and 2-E-E tend to have higher G′ and G″ values than that of −10 °C, indicating the more obvious positive effect of LFE field-assisted freezing. As shown in Figure 4(C1), the 3-E-N and 3-E-E groups have relatively higher G′ values between 1 and 10 Hz, suggesting the LFE field-assisted freezing helps maintain the elasticity of freeze–thawed cheeses. The 3-E-M also showed a higher G′ value between 2 and 10 Hz. The cheese of the 3-N-N group has the lowest G′ value under the same conditions. It can be seen from Figure 4(C2) that 3-N-N showed a similar G″ value to fresh cheese at low frequency (<4 Hz). All freeze–thawed samples showed closer G″ values at a higher frequency. However, it should be noted that 3-E-N and 3-E-E groups kept higher G″ values than other groups over 5 Hz, indicating better viscosity of cheese. It was reported that there was no significant difference in terms of rheological behavior between frozen samples or storage in different low temperatures [18]. Based on the above results, it can be concluded that the LFE field has a slight effect on the dynamic viscoelastic behavior of freeze–thawed cheese at low frequency and relatively higher freezing temperature. However, the LFE field-assisted frozen cheeses at −30 °C have higher elasticity.

### 3.4. Texture Profile Analysis

The texture profile analysis (TPA) results of the cheeses are shown in Table 3. The reversibility of all freeze–thawed cheeses is significantly lower than the fresh cheese due to the different degrees of damage and the low-temperature denaturation of protein. Studies have shown that ultra-low-temperature freezing can change the secondary and tertiary structure of a protein, and then affect the quality of fish [19]. Generally speaking, the stronger the structural integrity, the higher the recovery. However, this result is inconsistent with the previous conclusion on the degree of structural damage, which may be related to the low-temperature denaturation of protein. The reversibility of all frozen groups showed no significant difference. All cheeses retained similar cohesion to the fresh cheese. The LFE field-assisted freezing cheese has significantly lower adhesion and chewiness than the CK group, but a significantly higher adhesion and chewiness than the other treatments. Hardness and elasticity are the main concerns of cheese. Compared with the fresh cheese, cheese of the 1-E-E group kept a hardness of 10,325 g, and showed no significant difference from the fresh one (*p* > 0.05). Additionally, the 1-E-N, 1-E-M, and 1-E-E groups have high elasticity close to fresh cheese. With the decrease in freezing temperature, all data of −20 and −30 °C showed a similar trend to that of −10 °C. It has been reported that freeze–thawed tofu became harder, more elastic, more sticky, and more cohesive with freeze–thaw treatment [20]. The frozen–thawed cheese of the 3-E-E group has closer chewiness, hardness, and elasticity to the fresh cheese, indicating a positive effect on the textural property under lower freezing temperatures [21].

### 3.5. Amino Acid Analysis

**Figure 5:**
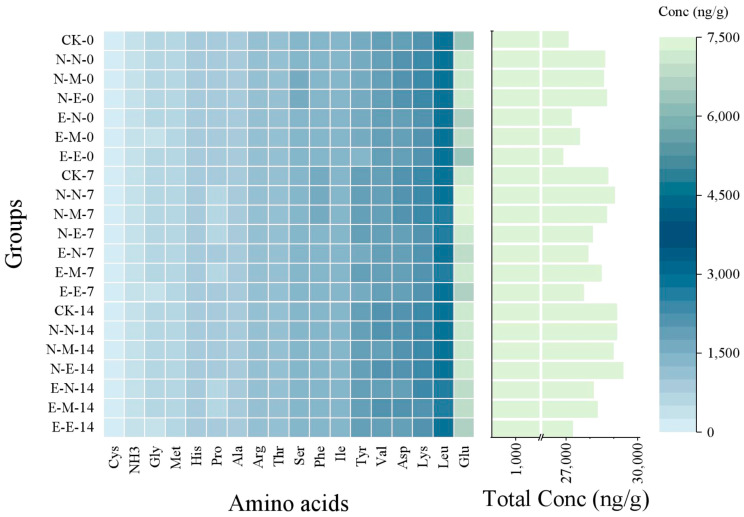
Amino acid of freeze–thawed Mongolian cheese after 30 days of storage.

The free amino acids and small peptides produced by the degradation of casein are crucial to the formation of cheese’s unique flavor, and this process can also affect the texture of cheese. The formation of ice crystals will change the matrix structure and affect the degradation reaction [22]. The free amino acids in cheese on the 0th, 7th, and 14th day during frozen storage at −30 °C are shown in Figure 5 and Table S2. It can be seen that the cheeses of the E-N-0 and E-E-0 groups have similar amino acid content to that of CK, but lower than that of other groups on the 0th day. In addition, the E-M-0 group also showed slightly higher amino acid content than that of CK, but lower than the N-N-0, N-E-0, and N-M-0 groups. This may be because big ice crystals disrupt protein structure and promote the low-temperature denaturation of protein [23]. The LFE field-assisted frozen cheeses retained a more stable protein structure, leading to less denaturation of protein. This may reduce the opportunity for the amino acid chain to be hydrolyzed, which is associated with lower free amino acid content. However, the heating process of microwave thawing affected the stability of the protein in the E-M-0 group. Similarly, denatured proteins expose more sites for hydrolase action, which may be the main reason for their higher free amino acid content than that of the CK group [24,25]. With the extension of storage time, the total amount of free amino acids of all groups showed an upward trend, indicating the normal post-ripening behavior of cheese under frozen storage [26]. Casein is considered to be a naturally denatured protein, which also plays an important role in the natural fermentation and solidification of cheese. The denaturation of proteins in cheese during storage can lead to changes in the secondary and tertiary structures and many groups of amino acid chains inevitably fall off and become free amino acids during this process [26].

The total amount of free amino acids in the CK group increased from 27,088.0 to 29,125.2 ng/g on the 14th day of storage. The increase of free amino acids in E-N, E-M, and E-E groups was slower than all the other groups during the 14 days of storage. The cheese of the E-E group only has the lowest free amino acid content of about 27,281 ng/g on the 14th day, indicating a slow proteolysis process of cheese during the storage period under this condition. In addition, the total amount of amino acids in microdefrosted cheese kept about 5~10% higher free amino acids content than that of other groups during the 14 days of storage. High free amino acids were always accompanied by the production of biogenic amines [27], leading to a bad sensory quality of cheese. In addition, the Arg amino acid is related to the formation of a bitter taste. The Arg amino acid is slightly lower in E-N, E-M, and E-E groups than in other frozen groups during the whole storage period. 

**Figure 6:**
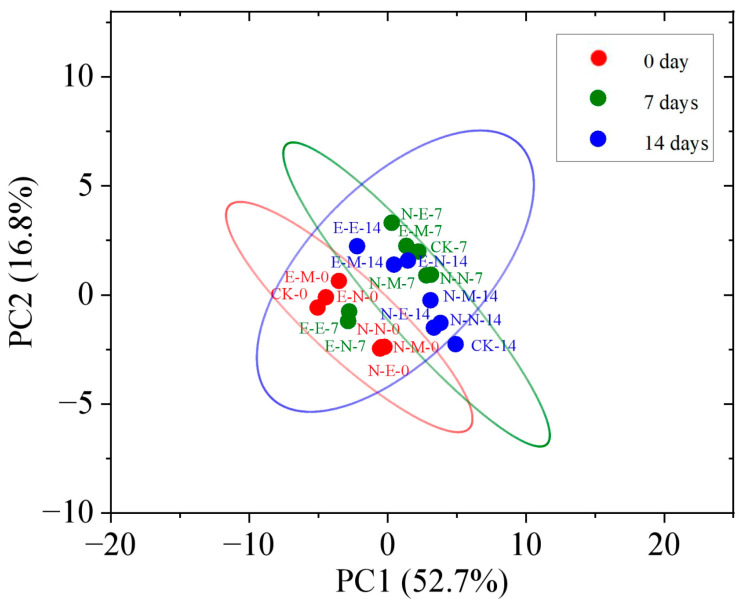
PCA analysis results of amino acid.

Principal component analysis (PCA) is applied to visualize the distribution of free amino acids in freeze–thawed cheeses with different treatments. Taking the storage time as a group, two principal components (PC) produced by 21 samples can explain 69.5% of the data-set variation (PC1 52.7% and PC2 16.8%) in Figure 6. PC1 and PC2 outlined two major groups, one group corresponding to cheeses on the 0th day and the other corresponding to cheeses on the 14th day. However, cheeses stored by the 7th day formed three disperse groups, which corresponded well to E-E-7, E-N-7, and other groups. E-E-7 and E-N-7 are in the confidence ellipse of the 0th-day group, while the points assigned to E-M-7 are in the confidence ellipse of the 14th-day group. In addition, it can be observed that the points ascribed to LFE field-assisted freezing or thawing samples are almost diagonally separated from other groups. The points of LFE field-assisted freeze–thawing groups on the 14th day are concentrated near the point of untreated groups on the 7th day, indicating that the involvement of the LFE field in freezing slowed down the transformation of amino acids. However, microwave thawing accelerated the proteolysis of cheese.

### 3.6. Fatty Acid Analysis

**Figure 7:**
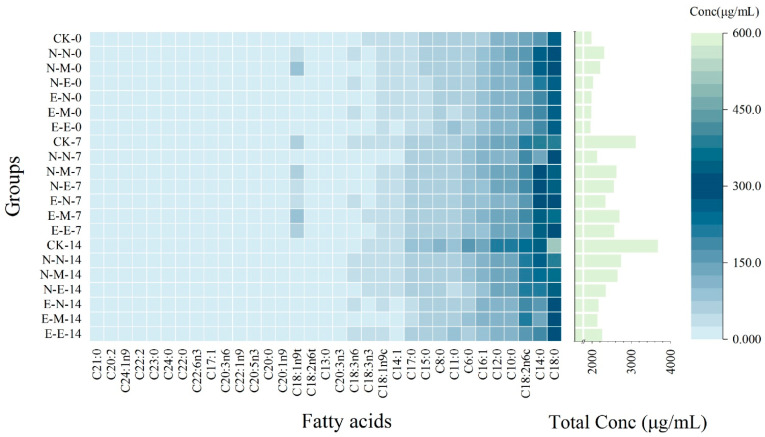
Fatty acid of freeze–thawed Mongolian cheese after 30 days of storage at −30 °C.

Fat flavor and acidity are closely related to the content of short-chain fatty acids [28]. In milk and dairy products, even-numbered fatty acids, such as butyric acid and lauric acid, can lead to a rancid flavor [29]. The changes in fatty acids in freeze–thawed cheese during the storage period are listed in Figure 7 and Table S3. Among the 36 kinds of common fatty acids in dairy products, C4:0 and C15:1 were not detected in cheese. The content of C16:0 is the highest. As can be seen from Figure 7, the N-N-0 and N-M-0 groups showed higher fatty acid contents than other groups at the beginning of storage. However, the N-E-0, E-N-0, E-M, and E-E-0 groups showed close or slightly lower fatty acids than the CK group. The total amount of free fatty acids in all cheese increased with the extension of storage time due to ripeness. It can be observed that the total free fatty acids of the CK group increased rapidly to 3675 μg/mL on the 14th day. However, the total free fatty acids of E-N-14, E-M-14, and E-E-14 groups were in the range of 2133 to 2251 μg/mL on the 14th day, indicating a slower ripening process. 

**Figure 8:**
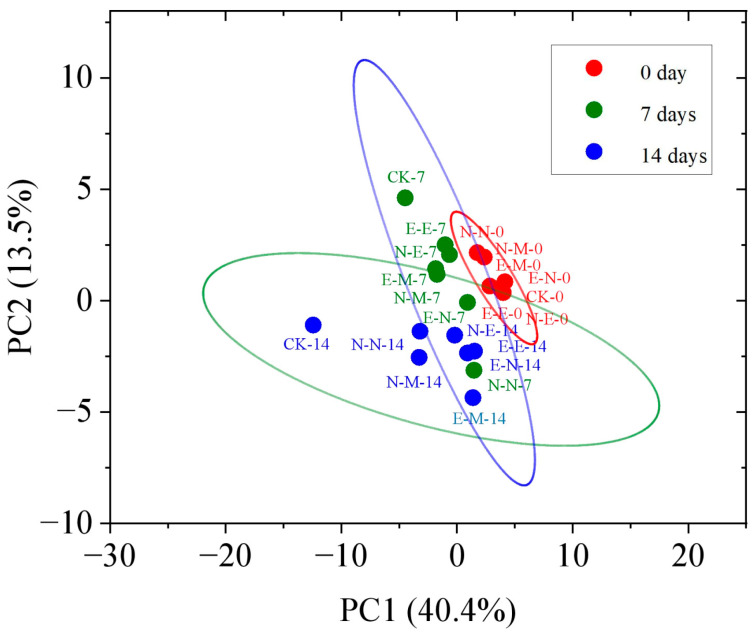
PCA analysis results of Fatty acid.

Taking the storage time as the grouping, two principal components (PC) generated from 21 samples can explain 52.9% of the data-set variation in Figure 8. From the perspective of fatty acid composition, samples with different storage times showed good separation. It is worth noting that the point of all LFE field-assisted frozen samples are closer to the group points of a sample on the 0th day. The sample of the N-N-7 group showed a better correlation with the group stored for 14 days. This indicates that the fatty acids in cheese without any treatment change faster than that of LFE field-assisted frozen samples. 

## 4. Summary

In this study, the sensory quality, microstructure, texture, and rheological properties of Mongolian cheese freeze–thawed under variable conditions were evaluated. Results showed that an LFE field can maintain freeze–thawed cheese with better microstructure and texture quality by reducing the damage of ice crystals. In addition, amino acids and fatty acids of the LFE field-assisted frozen cheese increased more slowly due to the delay of ripeness over 14 days of storage at −30 °C. An LFE field plays a positive role in the frozen storage of Mongolian cheese, suggesting a potential application in the freezing preservation of other dairy products.

## Figures and Tables

**Table 1:** Frozen–thawed orthogonal experimental design table.

Factors	A Frozen Temperature (°C)	B Thawing Temperature (°C)	C LFE Field
1	−10 °C	25	Frozen and thawing
2	−20 °C	Microwave	Frozen only
3	−30 °C		Thawing only

**Table 2:** Sample coding explanation.

Sample		Factors		
	Frozen Temperature	LFE Field-Assisted Frozen	Microwave Assisted Thawing	LFE Field-Assisted Thawing
Control group	4 °C	-	-	-
(1,2,3)-N-N	−10 °C	-	-	-
(1,2,3)-N-M	−10 °C	-	+	-
(1,2,3)-N-E	−10 °C	-	-	+
(1,2,3)-E-N	−10 °C	+	-	-
(1,2,3)-E-M	−10 °C	+	+	-
(1,2,3)-E-E	−10 °C	+	-	+

Note: “1,2,3” means frozen at −10, −20, or −30 °C; N means samples that were naturally frozen or thawed without any treatments; E means LFE field-assisted; M means microwave-assisted. “+” presents samples treated with this method, and “-”means no treatment.

**Table 3:** Texture properties of Mongolian cheese.

Sample	Hardness (g)	Elastic (J/F)	Cohesion	Adhesion (g·s)	Chewiness (g)	Reversibility	Moisture (g/100 g)
CK	10,695 ± 707 ^a^	0.85 ± 0.06 ^bcde^	0.24 ± 0.02 ^abcd^	2045 ± 100 ^b^	1720 ± 55 ^a^	0.93 ± 0.04 ^a^	0.56 ± 0.01 ^a^
1-N-N	6269 ± 146 ^i^	0.68 ± 0.05 ^h^	0.22 ± 0.03 ^bcdef^	1399 ± 167 ^e^	950 ± 76 ^f^	0.09 ± 0.01 ^bcd^	0.48 ± 0.01 ^ghi^
1-N-M	7556 ± 286 ^gh^	0.68 ± 0.03 ^h^	0.26 ± 0.04 ^a^	1500 ± 69 ^e^	970 ± 29 ^f^	0.10 ± 0.00 ^bc^	0.47 ± 0.01 ^hi^
1-N-E	6989 ± 413 ^hi^	0.73 ± 0.01 ^gh^	0.23 ± 0.01 ^abcde^	1605 ± 21 ^de^	1170 ± 10 ^e^	0.10 ± 0.01 ^bc^	0.51 ± 0.01 ^ef^
1-E-N	8546 ± 434 ^def^	0.84 ± 0.01 ^bcde^	0.18 ± 0.03 ^gh^	1914 ± 164 ^bc^	1482 ± 77 ^d^	0.09 ± 0.01 ^bc^	0.54 ± 0.01 ^bcd^
1-E-M	9497 ± 311 ^bc^	0.87 ± 0.05 ^abcd^	0.20 ± 0.01 ^fgh^	1936 ± 77 ^bc^	1577 ± 43 ^bcd^	0.10 ± 0.00 ^bc^	0.51 ± 0.00 ^ef^
1-E-E	10,325 ± 460 ^a^	0.80 ± 0.03 ^ef^	0.26 ± 0.03 ^a^	2567 ± 292 ^a^	1576 ± 25 ^bcd^	0.10 ± 0.01 ^b^	0.54 ± 0.00 ^abc^
2-N-N	7804 ± 361 ^fg^	0.87 ± 0.02 ^ab^	0.20 ± 0.01 ^fgh^	1480 ± 132 ^e^	1180 ± 45 ^e^	0.09 ± 0.00 ^bcde^	0.48 ± 0.02 ^ghi^
2-N-M	8609 ± 99 ^def^	0.91 ± 0.05 ^a^	0.23 ± 0.01 ^abcdef^	1533 ± 41 ^e^	1187 ± 44 ^e^	0.10 ± 0.01 ^bc^	0.46 ± 0.02 ^i^
2-N-E	8394 ± 473 ^ef^	0.82 ± 0.04 ^bcdef^	0.21 ± 0.01 ^efgh^	1756 ± 84 ^cd^	1218 ± 44 ^e^	0.08 ± 0.00 ^cde^	0.53 ± 0.02 ^cd^
2-E-N	9220 ± 380 ^cd^	0.81 ± 0.01 ^def^	0.22 ± 0.02 ^cdefg^	1998 ± 212 ^b^	1615 ± 160 ^abcd^	0.09 ± 0.01 ^bcde^	0.54 ± 0.00 ^bc^
2-E-M	9946 ± 644 ^abc^	0.83 ± 0.04 ^bcdef^	0.21 ± 0.03 ^defgh^	2005 ± 45 ^b^	1656 ± 82 ^abc^	0.10 ± 0.00 ^bc^	0.50 ± 0.00 ^fg^
2-E-E	10,679 ± 724 ^a^	0.86 ± 0.01 ^abcd^	0.26 ± 0.02 ^ab^	2623 ± 99 ^a^	1672 ± 147 ^abc^	0.10 ± 0.01 ^bc^	0.55 ± 0.01 ^abc^
3-N-N	8193 ± 394 ^efg^	0.81 ± 0.06 ^ef^	0.18 ± 0.01 ^h^	1416 ± 44 ^e^	1139 ± 101 ^e^	0.08 ± 0.00 ^de^	0.48 ± 0.01 ^gh^
3-N-M	9423 ± 596 ^bc^	0.81 ± 0.01 ^def^	0.22 ± 0.013 ^cdef^	1558 ± 148 ^de^	1267 ± 118 ^e^	0.07 ± 0.01 ^e^	0.48 ± 0.01 ^ghi^
3-N-E	9482 ± 345 ^bc^	0.81 ± 0.03 ^cdef^	0.20 ± 0.01 ^efgh^	1530 ± 13 ^e^	1215 ± 82 ^e^	0.09 ± 0.00 ^cde^	0.53 ± 0.02 ^cd^
3-E-N	10,158 ± 521 ^abc^	0.78 ± 0.01 ^fg^	0.19 ± 0.00 ^gh^	2015 ± 135 ^b^	1567 ± 84 ^cd^	0.08 ± 0.00 ^cde^	0.55 ± 0.00 ^abc^
3-E-M	10,330 ± 507 ^a^	0.82 ± 0.01 ^bcdef^	0.18 ± 0.01 ^h^	2079 ± 15 ^b^	1689 ± 43 ^abc^	0.09 ± 0.00 ^bcd^	0.52 ± 0.01 ^de^
3-E-E	10,533 ± 710 ^a^	0.87 ± 0.01 ^abc^	0.25 ± 0.01 ^abc^	2495 ± 173 ^a^	1711 ± 90 ^ab^	0.09 ± 0.01 ^bc^	0.55 ± 0.02 ^ab^

Note: The same superscript letters in the same column present no statistical significance (0.05); “1, 2, 3” means frozen at −10, −20, or −30 °C; N presents samples were naturally frozen or thawed without any treatments; E presents LFE field-assisted; M presents microwave-assisted.

## Data Availability

The data presented in this study are available on request from the corresponding author.

## Supplementary Materials

The following supporting information can be downloaded at: https://www.mdpi.com/article/10.3390/foods12081567/s1, Table S1: Sensory Scoring Criteria; Table S2: Free amino acid in Mongolian cheese on the 0th, 7th, and 14th day; Table S3. Free fatty acid in Mongolian cheese; Table S4. Parameters in regression equation of rheological properties.
