# Supplementary material for: Effects of Low-Frequency Electromagnetic Field on the Physicochemical Properties of Freeze–Thawed Mongolian Cheese
Source: Foods. 2023 Apr 7;12(8):1567. doi: 10.3390/foods12081567 (PMC10137472; doi:10.3390/foods12081567)
Supplement: Supplementary file 1 [file foods-12-01567-s001.zip › foods-2256070-supplementary.pdf]

Supplementary Materials: The following supporting information can be downloaded at: www.mdpi.com/xxx/s1, Table S1: Sensory Scoring Criteria; Table S2：Free amino acid in Mongolian cheese on 0, 7th and 14th day; Table S3. Free fatty acid in Mongolian cheese.Table S4. Parameters in regression equation of rheological properties

Table S1. Sensory Scoring Criteria

Sensory terms	Definition	Scores
Appearance (20 points)	Uniform glossy Milky white	≥15
	Uniform Milky white	9~14
	Lusterless light grey color	≤8
Smell (20 points)	Strong milk flavor with normal light fermentation sour	≥15
	Mild milk flavor with sour	9~14
	Light milk flavor or no flavor with pungent sour or odor	≤8
Taste (25 points)	Strong milk flavor with normal light fermentation sour	≥20
	Light milk flavor with obvious sour	10~19
	No milk flavor, bitter and over sour	≤9
Sensory texture (35 points)	Uniform smooth texture, semi-soft and elastic	≥30
	Basically uniform texture, slightly hard, finer tissue	11~29
	Rough texture, hard, fragile	≤10

Table S2. Free amino acid in Mongolian cheese on 0, 7th and 14th day.

Groups	Asp	Thr	Ser	Glu	Gly	Ala	Cys	Val	Met	Ile	Leu	Tyr	Phe	Lys	NH_3	His	Arg	Pro	Total (ng/g)
E-E-0	1886	1065	1285	6364	559	870	41	1880	603	1348	2861	1491	1344	2204	446	792	1036	796	26871
E-N-0	1933	1072	1331	6532	549	865	45	1867	597	1335	2852	1574	1395	2200	491	776	1038	774	27225
E-M-0	1915	1098	1461	6862	497	883	45	1841	562	1320	2844	1580	1414	2196	477	774	1032	783	27585
N-E-0	2027	1131	1511	7196	518	926	49	1915	556	1361	2968	1616	1480	2278	496	803	1069	817	28714
N-N-0	2026	1115	1475	7120	514	915	58	1928	576	1375	2971	1623	1476	2279	500	807	1070	808	28635
N-M-0	2041	1137	1501	7085	518	919	60	1914	565	1369	2951	1612	1463	2262	494	801	1064	822	28577
CK-0	1899	1078	1310	6464	568	865	34	1880	604	1347	2858	1532	1376	2187	489	780	1022	795	27088
E-E-7	1959	1122	1393	6638	500	888	67	1898	672	1363	2904	1593	1400	2227	489	792	1048	787	27739
E-N-7	1938	1115	1469	6879	506	899	77	1865	623	1335	2884	1602	1423	2208	476	783	1043	800	27925
E-M-7	1989	1130	1483	7232	512	932	40	1878	551	1376	2709	1856	1482	2249	424	807	1090	739	28479
N-E-7	1973	1121	1446	7169	503	919	37	1851	516	1350	2687	1824	1476	2227	419	795	1069	727	28109
N-N-7	2037	1158	1486	7388	516	947	43	1916	577	1407	2768	1882	1515	2300	427	824	1108	744	29042
N-M-7	2012	1137	1474	7321	508	931	39	1890	517	1382	2749	1872	1511	2279	426	813	1103	735	28702
CK-7	2057	1155	1476	7176	521	944	36	1917	534	1424	2759	1859	1503	2289	430	826	1093	754	28755
E-E-14	1886	1083	1265	6508	497	924	48	1982	611	1380	2750	1754	1367	2201	426	799	1049	753	27281
E-N-14	1964	1140	1376	6920	511	922	81	1915	634	1414	2724	1817	1432	2251	425	819	1073	743	28161
E-M-14	2005	1146	1439	6964	511	922	46	2020	561	1416	2717	1815	1464	2248	424	812	1070	739	28321
N-E-14	2065	1221	1460	7140	550	958	27	2028	587	1494	2911	1863	1484	2411	448	839	1152	766	29405
N-N-14	2033	1214	1449	7165	546	953	24	2003	584	1476	2868	1830	1461	2370	442	827	1129	759	29132
N-M-14	2035	1171	1458	7149	527	949	55	1945	653	1441	2782	1894	1490	2305	428	833	1114	762	28993
CK-14	2036	1170	1398	7126	532	949	112	2068	643	1454	2817	1851	1475	2333	436	845	1110	769	29125

Table S3. Free fatty acid in Mongolian cheese

Groups	E-E0	E-N0	E-M0	N-E0	N-N0	N-M0	CK0	E-E7	E-N7	E-M7	N-E7	N-N7	N-M7	CK7	E-E14	E-N14	E-M14	N-E14	N-N14	N-M14	CK14
C6:0	52	56	60	57	74	56	71	87	75	86	85	78	92	96	64	63	78	70	93	91	152
C8:0	73	54	52	54	64	54	58	67	60	67	67	63	67	71	56	61	63	63	73	71	101
C10:0	150	109	105	111	128	110	118	139	123	140	136	124	135	145	122	123	124	130	150	144	210
C11:0	69	72	48	69	68	64	60	74	70	68	72	74	68	61	78	75	74	42	69	52	99
C12:0	147	106	105	108	122	108	105	133	119	136	130	119	131	141	124	121	119	127	147	141	204
C13:0	14	12	13	13	14	13	15	14	12	15	13	14	14	15	7	13	7	14	14	14	18
C14:0	262	189	193	206	256	251	173	326	290	334	319	125	319	379	189	157	125	219	262	250	349
C14:1	32	25	26	28	35	34	42	40	36	41	39	15	40	44	23	19	15	27	32	30	37
C15:0	67	48	50	48	54	50	52	60	55	61	58	53	60	67	56	54	53	57	67	64	89
C16:0	843	586	580	589	663	641	570	705	658	775	733	648	738	817	690	644	648	712	843	811	1178
C16:1	110	80	81	84	96	90	75	98	92	100	99	100	105	113	94	101	100	95	110	109	147
C17:0	58	44	44	44	46	46	43	50	53	55	52	54	55	256	51	47	48	52	58	58	80
C17:1	6	6	11	12	14	7	6	16	6	15	17	8	16	20	7	6	8	7	6	6	7
C18:0	386	270	274	272	292	294	254	323	300	367	336	305	332	391	312	285	305	326	386	367	524
C18:1n9t	19	15	9	8	41	78	11	72	43	91	47	11	56	64	18	17	11	14	19	20	14
C18:1n9c	34	26	26	28	29	37	32	35	27	30	29	15	35	39	31	26	15	33	34	33	47
C18:2n6t	14	11	12	12	12	12	12	14	13	13	13	12	14	14	14	12	12	13	14	14	19
C18:2n6c	206	144	151	144	155	153	141	174	161	176	182	186	196	221	167	186	206	216	206	216	246
C20:0	9	8	6	8	5	8	8	8	8	9	9	9	9	9	8	8	9	9	9	9	9
C18:3n6	26	18	30	27	30	6	22	8	30	7	6	7	25	32	28	31	7	7	26	28	10
C18:3n3	28	22	22	22	22	22	26	24	23	27	24	23	25	31	30	22	23	25	28	27	38
C20:1n9	12	11	10	10	10	9	9	9	7	9	9	8	9	11	10	8	8	9	12	9	13
C21:0	3	3	3	3	3	3	3	3	3	3	3	3	3	3	3	3	3	3	3	3	4
C20:2	5	2	2	3	3	2	4	5	3	3	1	1	1	2	2	4	1	3	5	3	1
C22:0	5	6	6	6	6	6	6	6	6	6	5	4	6	6	4	6	4	5	5	7	7
C20:3n6	6	8	4	3	11	5	6	13	8	6	13	9	6	16	5	11	9	9	6	6	8
C20:3n3	17	15	14	15	16	3	15	16	15	18	15	15	16	3	17	16	15	17	17	19	23
C22:1n9	6	7	7	7	6	6	6	6	6	6	6	7	6	6	7	6	7	5	6	5	5
C23:0	6	6	5	4	6	6	5	6	6	6	6	6	5	7	6	4	6	6	6	5	7
C22:2	5	4	4	4	4	5	5	5	5	5	5	5	5	5	4	4	5	5	5	4	4
C20:5n3	6	6	6	6	6	7	8	6	7	7	5	7	8	6	7	10	7	5	6	11	9
C24:0	6	5	5	6	5	5	5	5	6	6	5	7	6	6	6	6	7	6	6	6	6

C24:1n9	5	5	5	4	5	6	5	4	6	4	5	4	5	5	5	5	4	4	5	5	5
C22:6n3	6	5	5	7	5	5	6	6	4	5	5	5	6	6	6	5	5	5	6	5	5
Total (μg / mL)	2734	1984	1974	2022	2304	2202	1978	2559	2335	2697	2548	2126	2614	3109	2251	2159	2133	2341	2734	2644	3675

Table S4. Parameters in regression equation of rheological properties

		CK	N-N	N-M	N-E	E-N	E-M	E-E
	Equation				y = Intercept + B1*x^1 + B2*x^2			
Figure A1	Intercept	169.53478 ± 15.37054	85.6455 ± 9.74121	105.41033 ± 15.80458	85.6435 ± 19.49317	73.55017 ± 10.09675	72.74033 ± 17.49276	78.747 ± 10.42204
	B1	-3.68436 ± 6.41939	-6.39581 ± 4.06834	-6.66286 ± 6.60066	-16.90036 ± 8.14117	-4.2799 ± 4.21683	-11.89786 ± 7.30572	-5.76392 ± 4.35268
	B2	1.59851 ± 0.56873	1.23458 ± 0.36044	1.73417 ± 0.58479	2.27458 ± 0.72128	1.32473 ± 0.3736	1.6528 ± 0.64726	1.15402 ± 0.38563
	RSS	1195.50016	480.17196	1263.97156	1922.80932	515.86313	1548.41737	549.63788
	COD	0.93532	0.91338	0.91879	0.80951	0.94933	0.75216	0.89461
	Adjusted	0.91684	0.88863	0.89558	0.75509	0.93486	0.68134	0.86449
Figure B1	Intercept	169.53478 ± 15.37054	79.869 ± 24.37425	93.684 ± 25.02421	95.683 ± 19.91358	107.982 ± 13.76328	87.81033 ± 18.02459	87.6335 ± 9.75092
	B1	-3.68436 ± 6.41939	-17.3625 ± 10.17971	-13.58482 ± 10.45116	-15.96671 ± 8.31675	-6.16603 ± 5.74813	-12.18014 ± 7.52783	-8.09127 ± 4.07239
	B2	1.59851 ± 0.56873	2.17023 ± 0.90188	2.18409 ± 0.92593	2.23371 ± 0.73683	1.51621 ± 0.50926	1.7878 ± 0.66694	1.57095 ± 0.3608
	RSS	1195.50016	3006.30921	3168.77881	2006.64176	958.55115	1644.00137	481.12983
	COD	0.93532	0.66558	0.78414	0.81331	0.91509	0.79333	0.94502
	Adjusted	0.91684	0.57003	0.72247	0.75997	0.89083	0.73428	0.92931
Figure C1	Intercept	169.53478 ± 15.37054	69.6385 ± 17.83345	88.04318 ± 10.96942	94.04848 ± 20.58067	105.19123 ± 13.1733	81.8509 ± 16.05276	105.62457 ± 13.36182
	B1	-3.68436 ± 6.41939	-11.83377 ± 7.448	-9.71794 ± 4.58129	-11.77255 ± 8.59536	-4.82014 ± 5.50173	-3.17554 ± 6.70431	-3.32468 ± 5.58046
	B2	1.59851 ± 0.56873	1.76663 ± 0.65987	1.44842 ± 0.40589	1.76473 ± 0.76152	1.80016 ± 0.48743	1.09017 ± 0.59398	1.67137 ± 0.49441
	RSS	1195.50016	1609.3191	608.88924	2143.33643	878.13371	1303.97917	903.44629
	COD	0.93532	0.79937	0.87581	0.75085	0.95839	0.84372	0.95716
	Adjusted	0.91684	0.74205	0.84033	0.67966	0.94651	0.79907	0.94492

Figure A2	Intercept	43.37009 ± 2.34484	23.28233 ± 1.55085	33.1805 ± 2.26491	27.608 ± 1.64126	22.21133 ± 2.02688	23.555 ± 2.057	21.05417 ± 1.73621
	B1	-0.10432 ± 0.9793	-0.10865 ± 0.6477	0.13393 ± 0.94592	-0.67697 ± 0.68546	-2.88779 ± 0.84651	-0.76538 ± 0.85909	0.41689 ± 0.72512
	B2	0.30643 ± 0.08676	0.20159 ± 0.05738	0.25966 ± 0.08381	0.25424 ± 0.06073	0.48682 ± 0.075	0.22129 ± 0.07611	0.10686 ± 0.06424
	RSS	27.82263	12.17064	25.95823	13.6309	20.78865	21.4112	15.25378
	COD	0.97095	0.96961	0.96752	0.96742	0.96792	0.92271	0.93381
	Adjusted	0.96265	0.96093	0.95824	0.95812	0.95876	0.90062	0.9149
Figure B2	Intercept	43.37009 ± 2.34484	28.29717 ± 2.39235	35.08267 ± 2.7502	26.25383 ± 2.55963	26.05833 ± 1.78378	25.9445 ± 1.93035	23.775 ± 2.48222
	B1	-0.10432 ± 0.9793	-1.74901 ± 0.99915	-0.87242 ± 1.1486	0.20111 ± 1.06901	-0.22041 ± 0.74498	-1.31255 ± 0.8062	0.39062 ± 1.03668
	B2	0.30643 ± 0.08676	0.30466 ± 0.08852	0.30667 ± 0.10176	0.19314 ± 0.09471	0.23189 ± 0.066	0.30269 ± 0.07143	0.16674 ± 0.09185
	RSS	27.82263	28.96153	38.27356	33.15314	16.10098	18.85574	31.17832
	COD	0.97095	0.90005	0.93663	0.93357	0.96731	0.9532	0.93136
	Adjusted	0.96265	0.87149	0.91852	0.91459	0.95797	0.93982	0.91174
Figure C2	Intercept	43.37009 ± 2.34484	42.2995 ± 2.57774	29.25487 ± 2.8881	27.47583 ± 2.39551	18.48233 ± 1.4325	29.25487 ± 2.8881	25.9615 ± 3.99027
	B1	-0.10432 ± 0.9793	-2.04323 ± 1.07657	0.67222 ± 1.20619	-0.01361 ± 1.00047	4.75056 ± 0.59827	0.67222 ± 1.20619	0.55295 ± 1.66651
	B2	0.30643 ± 0.08676	0.15746 ± 0.09538	0.09432 ± 0.10686	0.21042 ± 0.08864	-0.20144 ± 0.053	0.09432 ± 0.10686	0.20837 ± 0.14765
	RSS	27.82263	33.62398	42.20794	29.03799	10.38386	42.20794	80.57056
	COD	0.97095	0.38533	0.85348	0.94064	0.98152	0.85348	0.89553
	Adjusted	0.96265	0.20971	0.81161	0.92368	0.97624	0.81161	0.86569
